# Analysis of Soluble Protein Contents from the Nematocysts of a Model Sea Anemone Sheds Light on Venom Evolution

**DOI:** 10.1007/s10126-012-9491-y

**Published:** 2012-11-15

**Authors:** Yehu Moran, Daniela Praher, Ami Schlesinger, Ari Ayalon, Yossi Tal, Ulrich Technau

**Affiliations:** 1 Department of Molecular Evolution and Development, Centre for Organismal Systems Biology, Faculty of Life Sciences, University of Vienna, Althanstrasse 14, 1090 Vienna, Austria; 2 Maagan Michael; D.N., Menashe, 37805 Israel; 3 Golda Meir St. 54, Haifa, 34982 Israel; 4 Hamigdal St. 14, Caesarea, 30889 Israel

**Keywords:** Nematocyst, Toxin, Cnidaria, Nematostella, Venom

## Abstract

The nematocyst is one of the most complex intracellular structures found in nature and is the defining feature of the phylum Cnidaria (sea anemones, corals, jellyfish, and hydroids). This miniature stinging organelle contains and delivers venom into prey and foe yet little is known about its toxic components. In the present study, we identified by tandem mass spectrometry 20 proteins released upon discharge from the nematocyst of the model sea anemone *Nematostella vectensis*. The availability of genomic and transcriptomic data for this species enabled accurate identification and phylogenetic study of these components. Fourteen of these proteins could not be identified in other animals suggesting that they might be the products of taxonomically restricted genes, a finding which fits well their origin from a taxon-specific organelle. Further, we studied by in situ hybridization the localization of two of the transcripts encoding the putative nematocyst venom proteins: a metallopeptidase related to the Tolloid family and a cysteine-rich protein. Both transcripts were detected in nematocytes, which are the cells containing nematocysts, and the metallopeptidase was found also in pharyngeal gland cells. Our findings reveal for the first time the possible venom components of a sea anemone nematocyst and suggest their evolutionary origins.

**Electronic supplementary material:**

The online version of this article (doi:10.1007/s10126-012-9491-y) contains supplementary material, which is available to authorized users.

## Introduction

Cnidaria is a phylum that includes a wide variety of marine animals such as sea anemones, corals, jellyfish, and hydroids. All cnidarians are carnivores and utilize venom in order to catch their prey and defend themselves from predators. The defining hallmark of this animal group is the nematocyst, a highly complex proteinaceous structure made of a capsule containing an inverted tubule, capable of extremely fast and powerful discharge (David et al. 2008; Kass-Simon and Scappaticci 2002; Nuchter et al. 2006). Nematocysts are found inside cells called nematocytes, also known as stinging cells. These cells are considered to produce the toxins and their nematocysts are miniature injectors which deliver the venom into their prey or predator (Kass-Simon and Scappaticci 2002). However, only in few cases toxins were directly shown to reside in the nematocyst capsule (Hessinger et al. 1973; Honma et al. 2003; Lotan et al. 1995; Schlesinger et al. 2009).

Compared to other venomous animals such as snakes or scorpions, relatively little is known about cnidarian toxins and their biological activity. However, in contrast to the situation in other cnidarians, peptide toxins from sea anemones (Actiniaria) are relatively well studied. The vast majority of known toxins from sea anemones can be divided into three functional groups: (a) toxins modulating voltage-gated sodium channels (Moran et al. 2009; Wanke et al. 2009), (b) toxins which block or modulate voltage-gated potassium channels (Castaneda and Harvey 2009), and (c) cytolytic toxins disrupting membranes (Anderluh and Macek 2002).

The starlet anemone *Nematostella vectensis* has become a major model for the study of evolutionary developmental biology since unlike many other cnidarians it can be grown throughout its full life cycle in the lab and advanced molecular tools for its study are available (Darling et al. 2005; Technau and Steele 2011). These tools, including gene knockdown and transgenesis techniques (Nakanishi et al. 2012; Renfer et al. 2010; Technau and Steele 2011), put *Nematostella* in a unique position for the study of cnidarian toxin production and delivery. Moreover, in light of the recent emergence of sea anemone nematocysts as a potential drug delivery device (Ayalon et al. 2011), the ability to maintain large *Nematostella* cultures makes this species an attractive nematocyst source. However, despite its growing popularity as a lab model, until recently very little was known about its venom. A bioinformatic search of the *N. vectensis* genome sequence revealed that this species contains only one toxin homologous to previously described sea anemone toxins (Moran and Gurevitz 2006). The toxin, called Nv1, belongs to the type I sea anemone toxin group and like other members of this group it inhibits the activation of voltage-gated sodium channels, resulting in strong contractile paralysis and death of arthropods and fish (Moran et al. 2012b; Moran et al. 2008). Unexpectedly, Nv1 was localized to ectodermal gland cells in the tentacles rather than nematocysts and was shown to be released in massive amounts to the medium upon tentacle contact with prey (Moran et al. 2012b). These findings raise the question which venom proteins, if any, are produced by *N. vectensis* nematocytes. To answer this question, we have isolated nematocysts from *N. vectensis*, discharged them in vitro and analyzed the released protein mixture by tandem mass spectrometry (MS/MS). In addition to pointing out putative toxins and auxiliary venom proteins, the analysis uncovers intriguing evolutionary links between venom and non-venom proteins and reveals a collection of taxonomically restricted putative venom proteins.

## Materials and Methods

### Capsule Isolation and Discharge and Isolation of Secreted Proteins

Capsule isolation was carried out in a similar fashion to a previously published method (Zenkert et al. 2011). In brief, the whole 6-month-old *N. vectensis* polyps were frozen in liquid nitrogen and then homogenized in isolation solution (50 % Percoll, 10 % sucrose and 0.003 % Triton X-100). The mixture was then centrifuged at 2,000×*g*, 4 °C for 10 min.

Isolated *Nematostella* capsules were activated for 15 min by water. Discharge and capsule purity were verified under the microscope. The discharged suspension was centrifuged at 20,000×*g*, 4 °C for 10 min. The supernatant was lyophilized and sent to the Smoler Proteomics Center in the Technion (Israel Institute of Technology) for protein identification by tandem mass-spectrometry (MS/MS).

### Tandem Mass-Spectrometry (MS/MS) and Protein Annotation

The proteins from the samples were trypsinized and the tryptic peptides were analyzed by LC-MS/MS on the Orbitrap mass spectrometer (Thermo). The MS data was analyzed using the Sequest 3.31 software (Thermo) vs. the cnidaria section of the non-redundant protein sequences dataset (nr) of the National Center for Biotechnology Information (NCBI; http://blast.ncbi.nlm.nih.gov/Blast.cgi) and scores for each hit were calculated (Table S1). Conserved domains were detected using the CDD tool (Marchler-Bauer et al. 2011).

### RNA Isolation and Rapid Amplification of cDNA Ends (RACE)

Total RNA was isolated from 9-day-old primary polyps of *N. vectensis* using Trizol (Ambion, USA) according to the manufacturer’s instructions. The PolyA RNA was selected using the PolyATract mRNA isolation system III (Promega, USA). The isolated polyA RNA was used as template for all reverse transcription reactions performed. 5′ and 3′ RACE experiments were conducted using the RACE SMARTer kit (Clontech, USA) according to manufacturer’s instructions. Advantage2 DNA polymerase mix (Clontech) was used for PCR under the touchdown conditions suggested in the RACE SMARTer kit manual. The product of each initial PCR reaction in a final dilution of 1:1,000 served as template for a nested PCR. All primer sequences are available in Table S2. The PCR products were ligated into pGEM-T (Promega) and sequenced from both sides. The full transcripts encoding NEP-6 and NEP-16 were deposited in GenBank (Accession numbers JQ829079 and JQ829080).

### Phylogenetic Analysis

The boundaries of CAP and Astacin domains were determined according to PFAM. The domains were aligned using MUSCLE and for the SCP domains low quality alignment regions were removed by TrimAl (Capella-Gutierrez et al. 2009; Edgar 2004). ProtTest was used to find the most suitable model for phylogeny reconstruction (Abascal et al. 2005). For both domain alignments, the maximum-likelihood (ML) phylogenetic tree was constructed using PhyML with the WAG Model (+I + G), which got the highest score in the ProtTest analysis. Support values were calculated using 100 bootstrap replicates. A Bayesian tree was constructed using MrBayes version 3.1.2 with the same model. The run lasted 5,000,000 generations and every 100th generation was sampled. We estimated that the Bayesian analysis reached convergence when the potential scale reduction factor reached 1.0.

### Single and Double In Situ Hybridization (ISH)

For ISH experiments, *N. vectensis* larvae were fixed at 48–168 h post fertilization in ice-cold 3.7 % formaldehyde in 1/3 seawater with 0.2 % glutaraldehyde for 90 s and then in 3.7 % formaldehyde in 1/3 seawater without glutaraldehyde for additional 60 min. Transcript fragments were amplified by PCR and cloned into pGEM-T. Antisense RNA probes for ISH were generated and labeled by using the T7 or SP6 MEGAscript kits (Ambion) and an RNA labeling mix with either digoxygenin (DIG) or fluorescein (FITC; Roche, Germany). The ISH procedure for single probes was performed as described previously using DIG-labeled probes (Genikhovich and Technau 2009). For double in situ, a DIG-labeled and a FITC-labeled probe were hybridized simultaneously according to the single probe protocol (Genikhovich and Technau 2009). After the hybridization step and the following washes, sheep anti-FITC coupled to alkaline-phosphatase (Roche) was applied at a dilution of 1:2,000 in blocking reagent (Roche) and incubated overnight at 4 °C. The next morning, the samples were washed 10 times with phosphate buffer saline containing 0.1 % Tween-20 (PTw) and then were incubated in 0.1 M Tris–HCl (pH 8.2) for 5 min twice. Then FastRed reagent (Roche) was applied in the same buffer. After development of a strong red signal, the reaction was stopped by five quick washes followed by an inactivation of the enzyme by a single wash in 0.1 M glycine–HCl (pH 2.2) for 10 min at room temperature. After five additional washes in PTw, the samples were blocked for 1 h at room temperature with blocking reagent solution. Then sheep anti-DIG alkaline-phosphatase-coupled antibody (Roche) was applied at a concentration of 1:3,000 in blocking reagent and the samples were incubated overnight at 4 °C. The next morning the samples were washed ten times with PTw and nitroblue tetrazolium chloride (NBT)/5-bromo-4-chloro-3-indolyl-phosphate (BCIP) blue signal was developed as in the single in situ procedure (Genikhovich and Technau 2009). The staining was stopped by three washes in PTw and stained sampels were mounted either in SlowFade Gold medium (Invitrogen) or 85 % glycerol and photographed in a Nikon Eclipse 80i fluorescent microscope connected to a Nikon Digital Sight DS-U2 camera.

## Results and Discussion

### The Proteins Released from *Nematostella* Nematocysts Upon Discharge

In order to explore the possibility that venom proteins in *Nematostella* are produced by nematocytes and packed inside the nematocyst capsule, we isolated the soluble fraction of the nematocyst content that is released upon discharge and analyzed it by MS/MS. The measured peptides were automatically searched against the Cnidaria section of the non-redundant (nr) protein database provided by the NCBI. A clear indication for the accuracy of this method is that the best hit for 20 out of 23 peptide groups came from *Nematostella* genome-based protein models (Table 1 and Tabe S1). Moreover, manual search against the whole dataset and not specifically in the Cnidaria section did not change this result. The three non-*Nematostella* hits were probably an artifact of long glutamate polymers (Table S1) and may result from γ-glutamate polymers in the nematocyst lumen as was described for *Hydra* (Weber 1990). As expected, no peptide hits corresponded to the Nv1 toxin that was shown to be expressed only in gland cells (Moran et al. 2012b). We decided to name the 20 remaining proteins detected in the discharged mixture “Nematocyte expressed proteins” (NEPs) and numbered them according to the number of matching peptide fragments. Noticeably, most of them exhibited no strong homology to any characterized proteins, and some had no conserved domains at all. Among the clear homologues, we found two nematogalectin-like proteins (NEP-5 and NEP-9), two Tolloid-related proteins (NEP-6 and NEP-14) and a Cyclophilin-type peptidyl-prolyl *cis*–*trans* isomerase homologue (NEP-11). Nematogalectin is one of the components of the nematocyst capsule in hydrozoans and a conserved molecule in many cnidarians, including *Nematostella* (Hwang et al. 2010). The finding of two Nematogalectin homologues in our samples is surprising, since they are thought to be an integral part of the capsule matrix. While it is possible—in principle—that some capsule matrix contamination was present in the sample, we find this improbable, as in the MS/MS data there are no peptide hits for any other common nematocyst component such as Minicollagens (David et al. 2008; Zenkert et al. 2011). This suggests that Nematogalectins are also present as free monomers in the capsule lumen. Tolloid is a metallopeptidase of the Astacin family (EC 3.4.24.21) involved in developmental processes such as axis formation (see below). The Cyclophilin type peptidyl-prolyl *cis*–*trans* isomerases are enzymes (EC 5.2.1.8) that control the stability of other proteins and can serve as chaperones since they accelerate the isomerization of peptide bonds preceding a proline (Wang and Heitman 2005). The finding of such a protein inside the capsule raises the possibility it is an auxiliary venom component that stabilizes protein toxins. Since nematocysts might spend long time prior to discharge, stabilization of venom components by chaperones can be advantageous. Among the rest of the identified proteins, NEP-19 exhibits moderate sequence homology to Slit1b, a signaling molecule with roles in axon guidance and neuronal development in vertebrates (Chedotal 2007; Hutson et al. 2003). However, this homology is limited to a short amino acid stretch and might be spurious. Nine additional proteins do not exhibit homology to any characterized proteins from other animals, but do contain conserved domains (Table 1). Of these conserved domains, two were previously shown to be present in venom proteins of other animals: a chitin binding Peritrophin-A domain like that of NEP-1 was found in venom chitinases from wasps and the CAP domain (cysteine rich secretory protein [CRISP], antigen 5 [Ag5], and pathogenesis related 1 [PR-1]) was found in venom proteins from a large diversity of animal groups including snakes, spiders, cone-snails, and cephalopods (Fry et al. 2009a; Fry et al. 2009b; Krishnan et al. 1994).

**Table 1:** Proteins detected by tandem mass spectrometry in the secretion of discharged nematocysts of *Nematostella* and their homologies

Protein	No. of measured peptides	Best *Nemaostella* hit	Best non-model BLAST hit	Conserved domains and protein models
NEP-1	6	XP_001630235	–	2 Endoglucanase C-terminal domain(COG4305); Chitin binding Peritrophin-A domain (PF01607)
NEP-2	5	XP_001630639	–	RNA polymerase sigma-B factor (TIGR02941)
NEP-3	4	XP_001640559	–	–
NEP-4	4	XP_001638827	–	–
NEP-5	3	XP_001639343	BAJ22666; nematogalectin *Hydra vulgaris*	Galactose binding lectin domain (PF02140)
NEP-6	3	XP_001628963	ABC88377; Tolloid *Nematostella vectensis*	Astacin (PF01400)
NEP-7	2	XP_001621624	–	–
NEP-8	2	XP_001623695	–	–
NEP-9	2	XP_001639420	BAJ22673 nematogalectin related *Aurelia aurita*	Collagen triple helix repeat (PF01391). Galactose binding lectin domain (PF02140).
NEP-10	2	XP_001628965	–	–
NEP-11	2	XP_001626713	AAH59560 Ppib protein *Danio rerio*	Cyclophilin type peptidyl-prolyl *cis*–*trans* isomerase (PF00160)
NEP-12	1	XP_001629049	–	–
NEP-13	1	XP_001625592	–	Inosine-uridine preferring nucleoside hydrolase (PF01156)
NEP-14	1	XP_001623204	AAX98722 Tolkin *Drosophila simulans*	Astacin (PF01400)
NEP-15	1	XP_001632785	–	PLAT/LH2 (PF01477)
NEP-16	1	XP_001641525	–	CAP, Cysteine-rich secretory protein family (PF00188)
NEP-17	1	XP_001625851	–	CAP, Cysteine-rich secretory protein family (PF00188)
NEP-18	1	XP_001618722	–	Inosine-uridine preferring nucleoside hydrolase (PF01156)
NEP-19	1	XP_001625589	AAZ79235 Slit1b *Danio rerio*	2 EGF-like domains (PF00008)
NEP-20	1	XP_001626820	–	CAP, Cysteine-rich secretory protein family (PF00188)

### Putative Venom Metallopeptidases in *Nematostella* are Members of the Tolloid-Related Family

**Fig. 1:**
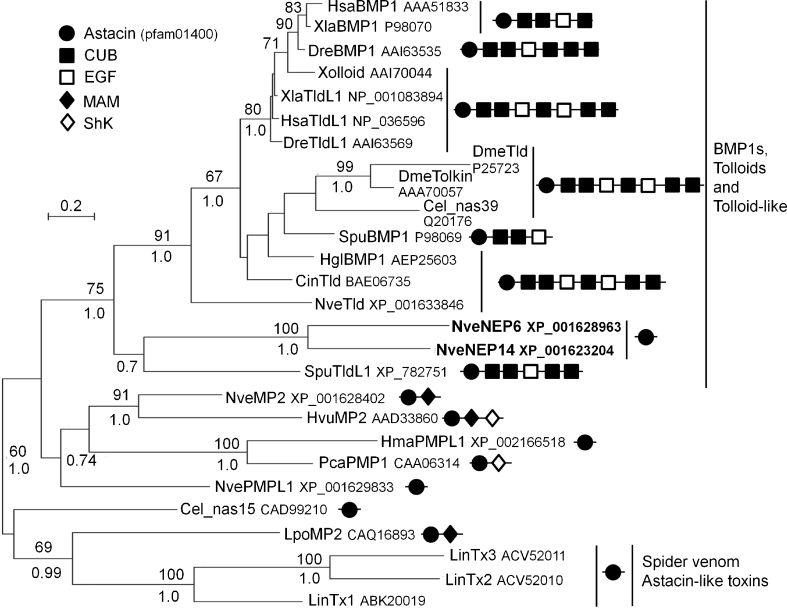
Phylogeny of proteins containing Astacin domains. A maximum likelihood unrooted phylogenetic tree was constructed with the WAG model (+I, +G). Bootstrap support values above 50 % are indicated above branches and Bayesian posterior probability values above 0.70 appear below branches. The proteins found in the *Nematostella* capsule are highlighted in *bold font*. Protein accession numbers (either GenBank or Swissprot) as well as conserved domains and their order appear to the right of the protein name. Abbreviations of protein names are: *BMP* bone morphogenic protein, *MP* metalloproteinase, *PMP Podocoryne* metalloproteinase, *PMPL Podocoryne* metalloproteinase-like, *NEP* nematocyst expressed protein, *Tld* tolloid, *TldL* tolloid-like, *Tx* toxin. Abbreviations of species names are: *Cel Caenorhabditis elegans* (nematode), *Cin Ciona intestinalis* (ascidian), *Dme Drosophila melanogaster* (fruit fly), *Dre Danio rerio* (zebrafish), *Hgl Holothuria glaberrima* (sea cucumber), *Hma Hydra magnipapillata* (hydra), *Hsa Homo sapiens* (human), *Hvu Hydra vulgaris* (hydra), *Lin Loxosceles intermedia* (brown spider), *Lpo Limulus polyphemus* (horseshoe crab), *Nve Nematostella vectensis* (sea anemone), *Pca Podocoryne carnea* (hydrozoan jellyfish), *Spu Strongylocentrotus purpuratus* (sea urchin), *Xla Xenopus laevis* (frog)

Two of the identified proteins, NEP-6 and NEP-14, contain an Astacin domain. Astacin domain proteins are secreted zinc-dependent metallopeptidases that cleave various extracellular targets. Unexpectedly, both *Nematostella* NEPs exhibit high similarity to Tolloid and its homologues. Tolloid (also known as BMP1, bone morphogenic protein 1) is conserved from cnidarians to vertebrates and plays a pivotal role in animal development by cleaving Chordin, the inhibitor of BMP2/4 (called Decapentaplegic, Dpp, in *Drosophila*; Marques et al. 1997; Piccolo et al. 1997). Dpp/BMP2/4 is a major player in establishing the dorso-ventral axis in arthropods and vertebrates and also plays a significant role in establishing axial asymmetries in *Nematostella* (Marques et al. 1997; Saina et al. 2009). In addition to activating Dpp via cleavage of Chordin, Tolloid controls further developmental pathways via cleaving other targets (Muir and Greenspan 2011). A typical Tolloid structure contains besides an N-terminal Astacin domain a long stretch of C-terminal CUB domains, which control its target specificity (Geach and Dale 2008; Fig. 1). Strikingly, both NEP-6 and NEP-14 have no CUB domains, as verified by 5′ and 3′ rapid amplification of cDNA ends (RACE; Fig. 1). This finding supports the idea that these metallopeptidases serve as toxins, since the absence of CUB domains diminishes their selectivity and makes them much more potent weapons. Other types of metallopeptidases are major components in the venom of vipers and cause hemorrhage as well as other types of substantial tissue damage (Kang et al. 2011). Strikingly, loss of additional domains in snake venom metalloproteases was shown to accelerate their evolution (Casewell et al. 2011). Up till now, Astacin-like proteins were found to act as toxins only in the venom of brown spiders (genus *Loxosceles)*, a group notorious for inducing lesions by their bite (da Silveira et al. 2007; Trevisan-Silva et al. 2010). A phylogenetic analysis shows that the astacin-domain of NEP-6 and NEP-14 clearly clusters with Tolloid, suggesting that they have derived from a tolloid-like ancestor molecule by loss of the CUB domain. By contrast, spider Astacin-like toxins cluster outside the BMP1/ Tolloid family (Fig. 1), indicating their independent recruitment to venom.

**Fig. 2:**
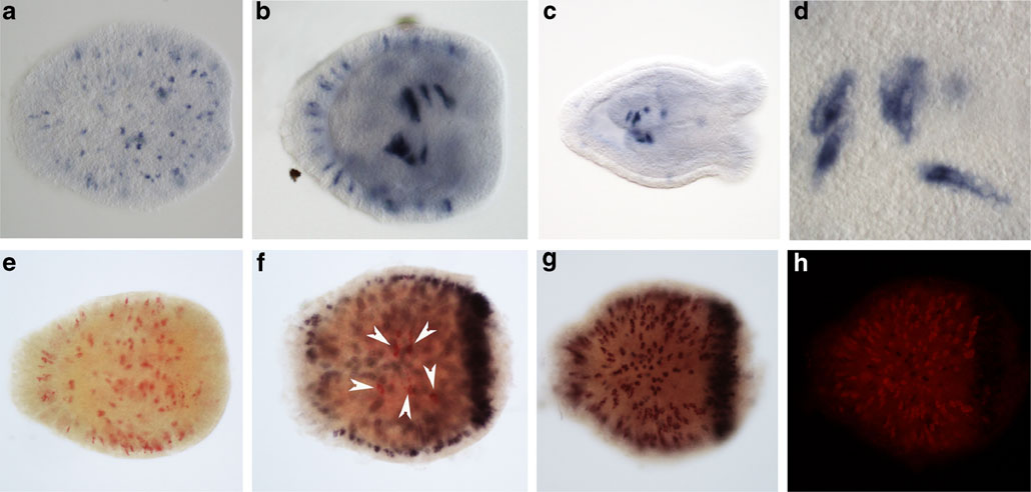
In situ hybridization (ISH) expression patterns of *NEP-6* in *Nematostella*. **a** Expression is restricted in a 2-day-old early planula to distinct long nematocyte-like ectodermal cells in the body wall (*dark blue* staining). **b** The expression domain expands in 3-day-old planulae and can be detected in few large cells in the developing pharynx in addition to the initial expression domain. **c** In primary polyps (7 days), the pharyngeal cells become the dominant domain of expression. **d** At this stage, *NEP-6* is mainly expressed in large pharyngeal cells (>30 μm), which probably are gland cells as indicated by unstained vesicles inside stained cells. Comparing *NEP-6* FastRed staining in single in situ **e** to double staining of *NEP-6* (*red*) and the nematocyte marker *NvNCol-3* (*dark brown*) **f** indicate that *NEP-6* is expressed in almost all *NvNCol-3* expressing nematocytes of the body wall ectoderm. In contrast, the *NEP-6* positive cells in the pharynx (red cells, indicated by *arrowheads*) do not express *NvNCol-3*. When the visible light **g** and red fluorescence (**h**, FastRed staining) images of a double-stained planula are compared, it is clear that some cells express *NvNCol-3* but not *NEP-6* (*dark cells*). In all pictures, the oral end of the animals is to the *right*

The *Nematostella tolloid* homologue (containing both CUB and astacin domains) was previously shown to be expressed mainly in the endoderm throughout the life cycle of the sea anemone, as was also observed in our experiments (Matus et al. 2006; Saina et al. 2009; Fig. S1). To verify that the Tolloid-related proteins resulting from our tandem mass spectrometry are restricted to nematocytes, we assayed the expression pattern of the NEP-6 transcript by ISH. NEP-6 is expressed in the early planula stage of *Nematostella* in single cells of the ectoderm. These cells are thin and elongated as would be expected of developing nematocytes (Fig. 2a). In the later stages, the expression expands to an additional domain of large thick cells in the pharynx (Fig. 2b, c). The size and richness of vesicles suggest that these cells are not nematocytes but gland cells, and in the polyp stage, they become the major expression domain of NEP-6 (Fig. 2c, d). Double ISH localizing *NEP-6* and the nematocyst minicollagen marker *NvNCol-3* simultaneously revealed that many *NEP-6* expressing cells in the suboral ectoderm are a subpopulation of minicollagen-expressing cells, i.e., in this region none of the body wall nematocytes express *NEP-6* without expressing *NvNCol-3* and only few cells express *NvNCol-3* but not *NEP-6* (Fig. 2e, g, h). By contrast, at the oral pole, large numbers of minicollagen-expressing nematocytes do not express *NEP-6*. In the pharynx however, the vesicle-rich cells expressing *NEP-6* do not express *NvNCol-3*, suggesting that they are not nematocytes (Fig. 2e–h). We propose that the pharyngeal expression of NEP-6 in gland cells is for killing and digesting swallowed prey, as was recently suggested for a pore-forming toxin homologue expressed in the same body region (Moran et al. 2012a). Expression of *NEP-6* in nematocysts seems to be mainly for defensive purposes, as it has no expression in the tentacle tips, which are used for catching prey.

### CAP Domains in the Putative Venom Proteins of *Nematostella*

**Fig. 3:**
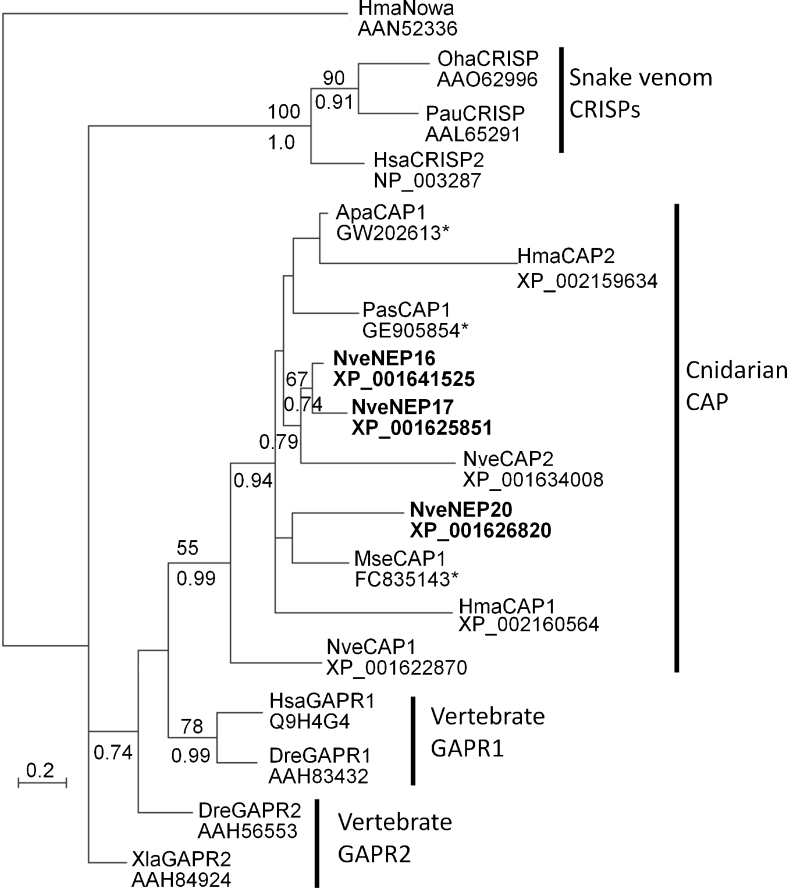
Phylogeny of proteins containing CAP domains. A maximum likelihood unrooted phylogenetic tree was constructed with the WAG model (+I, +G). Bootstrap support values above 50 % are indicated above branches and Bayesian posterior probability values above 0.70 appear below branches. The proteins secreted from the *Nematostella* nematocyst capsule are indicated by *bold font*. Protein accession numbers (either GenBank or Swissprot) appear to the right of the protein name. Protein sequences originating from translated expressed sequence tags are labeled with an *asterisk*. Abbreviations of protein names are: *CAP* cysteine rich secretory protein [CRISP], antigen 5 [Ag5], and pathogenesis related 1 [PR-1], *GAPR* Golgi-associated plant pathogenesis-related protein. Abbreviations of species names are: *Apa Acropora palmata* (stony coral), *Dre Danio rerio* (zebrafish), *Hma Hydra magnipapillata* (hydra), *Hsa Homo sapiens* (human), *Mse Metridium senile* (sea anemone), *Nve Nematostella vectensis* (sea anemone), *Oha Ophiophagus hannah* (elapid snake), *Pas Porites astreoides* (stony coral), *Pau Pseudechis australis* (elapid snake), *Xla Xenopus laevis* (frog)

**Fig. 4:**
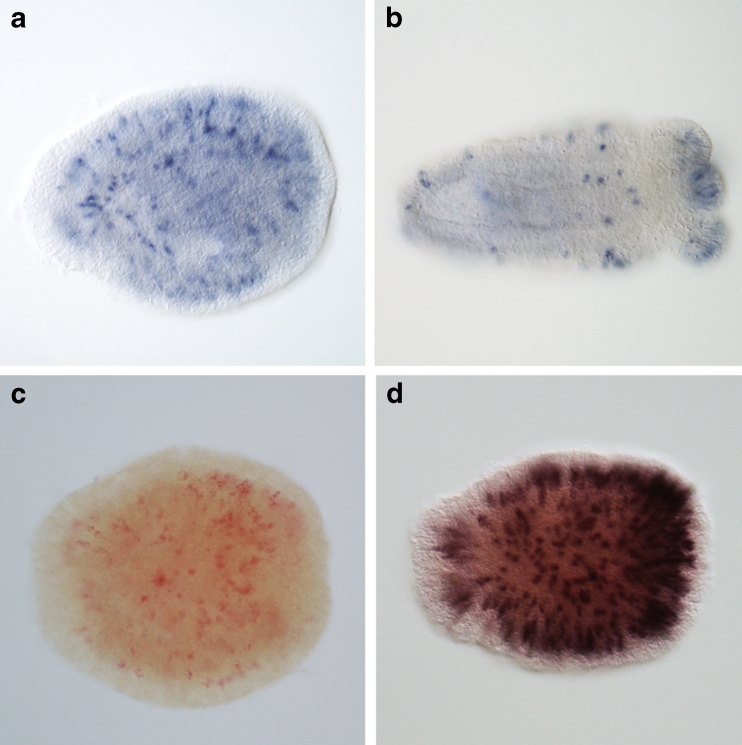
In situ hybridization (ISH) expression patterns of *NEP-16* in *Nematostella*. **a** Three-day-old planula. **b** Seven-day-old primary polyps. Note the distinct long nematocyte-like ectodermal cells in the body wall and tentacle tips (*dark blue* staining). **c** Single *NEP-16* expression stained with FastRed. **d** Double ISH of *NEP-16* and the nematocyst marker *NvNCol-3* (*dark brown–purple*) show perfect localization of *NEP-16* to nematocysts. In all pictures, the oral end of the animals lies to the *right*

NEP-16, -17, and -20 contain a CAP domain. This domain, also known as sperm coating glycoprotein (SCP) domain, is found in a wide variety of protein families from different kingdoms of life (Gibbs et al. 2008). Among CAP domain proteins, arguably the best studied are CRISPs, which are commonly found in mammalian male reproductive tract and snake venom. CRISPs were shown to be effective ion channel blockers, but this function stems from another small domain which is always located C-terminally of the CAP domain (Brown et al. 1999; Gibbs et al. 2008; Gibbs et al. 2006). However, this additional domain was not found in any non-CRISP CAP protein and is absent from NEP-16, -17, and -20. CAP domains were recently found in many proteins from *Hydra* nematocysts and as this domain is also present in a medusozoan-specific structural capsule protein called nematocyst outer wall antigen (NOWA), these proteins were suggested to have structural roles as well (Balasubramanian et al. 2012; Engel et al. 2002). However, we note that the CAP domain of NOWA is highly derived and does not cluster with any other cnidarian CAP protein (Fig. 3). Moreover, unlike NOWA and the majority of *Hydra* CAP-containing proteins, NEP-16, -17, and -20 and other *Nematostella* CAP proteins tend to be short and consist of only one CAP domain and no additional conserved domains (data not shown). Based on these observations, we do not see any strong indication that CAP domains in *Nematostella* carry structural roles. Interestingly, proteins very similar to NEP-16, -17, and -20 can be found in other anthozoans such as corals (Fig. 3). The fact that CAP domains are present in a large variety of venom proteins in other animals suggests that this domain can be involved in toxicity (Fry et al. 2009a; Fry et al. 2009b) and that NEP-16, -17, and -20 might be toxins. However, this suggestion remains to be tested experimentally. Interestingly, The CAP domain is more common in *Nematostella* than in vertebrates as we can detect 60 gene models containing CAP in the sea anemone genome compared to 31 in human and 33 in mouse (Gibbs et al. 2008). This observation raises the possibility that certain CAP proteins are nematocyst-specific. Indeed, the NEP-16 transcript is expressed exclusively in thin and long cells at the body wall ectoderm of the planula (Fig. 4a). In polyps the expression can also be detected in similarly shaped cells in the tentacle ectoderm (Fig. 4b). Localizing *NEP-16* and *NvNCol-3* simultaneously revealed that all cells expressing *NEP-16* also express *NvNCol-3*, thus proving their nematocyte identity (Fig. 4c, d).

### Convergent Toxin Recruitment Versus Novel Taxonomically Restricted Genes

In recent years with accumulation of data regarding venom components in various animals, it became clear that the same non-toxic protein families are re-currently becoming toxins via gene-duplication, accompanied by adaptive evolution and differential expression (Fry et al. 2009a; Fry et al. 2009b). The fact that the same gene families are recruited numerous times independently suggests that the non-toxic activity of these proteins can easily become toxic upon expression in a different context. Proteases and peptidases of all kinds can cause immediate tissue damage when lacking restraining selectivity, making them prime candidates for toxins in the venoms of diverse animal groups (Fry et al. 2009a). NEP-6 and NEP-14 are Astacin domain proteins that were probably recruited to the nematocyst merely due to their proteolytic activity. However, their clustering with Tolloid and Tolloid-like proteins suggest that their ancestor genes were involved in development (Fig. 1). The recruitment of Astacin-like proteins to spider venom and of many other non-Astacin peptidases to the venom of snakes demonstrates that the exact source of the proteolytic activity of venom is of little importance. In the cnidarian *Hydractinia echinata* an Astacin of another subfamily is expressed in developing nematocyte precursors (Mohrlen et al. 2006). Further, Astacins from *Hydra* were also shown to be present in the nematocyst capsule (Balasubramanian et al. 2012). It would be interesting to test whether these hydrozoan Astacins are secreted from the capsule upon discharge. A positive result will be pointing to a role as toxins and to an intriguing scenario where convergent recruitments of Astacin to venom occurred several times within Cnidaria.

Out of 20 proteins identified by MS/MS, 6 (30 %) do not show any clear homology to other proteins, and do not contain any conserved domains. Additional 8 proteins have at least 1 conserved domain but still do not exhibit profound homology (>25 % similarity) to any other metazoan protein currently present in the non-redundant protein database of the NCBI (Table 1). This means that in total 14/20 (70 %) of the proteins have no known homologue in another metazoan and may be considered as taxonomically restricted genes (TRGs; also known as orphan genes; Khalturin et al. 2009). This is a strong enrichment compared to results from recent re-annotation of the *Nematostella* transcriptome that found 16 % of the protein models to lack metazoan homologues and 5 % to lack both homologues and conserved domains (Fredman D. and Technau U. unpublished results). This is consistent with genetic studies in *Hydra*, indicating that a substantial fraction of the genes exclusively expressed in nematocytes are TRGs (Hwang et al. 2007; Milde et al. 2009). As the nematocyst is a unique cnidarian structure conserved for more than 600 million years it is plausible that many of its component proteins including toxins will be encoded by TRGs. Some nematocyst structural components, which are considered as TRGs such as Nematogalectins and Minicollagens, seem to be conserved among a wide range of cnidarians (David et al. 2008; Hwang et al. 2010), but as toxins are facing very strong selection due to prey-predator “arms race” their evolutionary turnover is usually much higher (Barlow et al. 2009; Duda and Palumbi 1999). CAP proteins in the *Nematostella* nematocyst lumen might represent a putative toxin class that is conserved in stony corals (Fig. 3), a remarkable feat when considering that the divergence time of stony corals and anemones is estimated at 500 million years ago (Shinzato et al. 2011). Thus, it is possible that CAP containing proteins represent an ancient toxin class whereas the other proteins we detected are newer venom recruits in the *Nematostella* lineage.

### Multiple Venom Sources in Sea Anemones

The localization of Nv1 and other type I toxins to ectodermal gland cells revealed a new venom producing cell population in sea anemones (Moran et al. 2012b). In the present work, we show evidence suggesting that nematocysts in *Nematostella* produce toxins of classes never described before for a sea anemone (Table 1). In the vast majority of past studies, toxins were purified from whole tentacles by harsh chemical extractions, which put in risk the structural integrity of many proteins. Thus, it is difficult to assess the completeness of the detected arsenal and to what degree nematocyst toxins were represented in the peptides studied in the last 37 years by activity-guided fractionation (Béress et al. 1975; Bruhn et al. 2001; Diochot et al. 2004; Peigneur et al. 2011; Schweitz et al. 1981; Turk and Kem 2009). It is possible that many more sea anemone toxins are waiting to be discovered by finer methods like water-induced discharge of isolated nematocysts. In a recent study, tentacles were treated by dipping in distilled water for discharging nematocysts, resulting in an unprecedented richness of toxin peptides from just two anemone species (Rodriguez et al. 2011). Nevertheless, we suggest this method is likely to release the content of toxin-producing gland cells as well, since these are ectodermal cells located on the very outer boundary of the anemone tentacle (Moran et al. 2012b).

It is currently unknown why certain toxins are secreted from nematocytes while others are produced in gland cells. However, it is clear that some toxins are produced by more than one cell population: the type I toxins of *Anemonia viridis* are produced in both ectodermal gland cells of the tentacle and nematocytes whereas NEP-6 of *Nematostella* is found in both body wall nematocysts and gland cells of the pharynx (Fig. 2; Moran et al. 2012b). The expression of the same toxin by both gland cells and nematocysts supports the theory that nematocysts evolved originally from toxin secreting gland cells (Balasubramanian et al. 2012; Moran et al. 2012b). The expression of toxins in *Nematostella* and probably in other cnidarian species seems to be complex as distinct cell types express peptide toxins in different parts of the animal in various life stages for diverse functions such as prey capture, prey disintegration, and defense (Moran et al. 2012b). How such a complex system is regulated and what are the unique features of each toxin producing cell type and its biochemical arsenal remains to be described.

## Conclusion

*Nematostella* nematocysts contain at least 20 proteins that are released upon capsule discharge. Some, like the Astacin domain metallopeptidases have a clear potential to act as potent toxins causing damage in stung prey or predator. Many others of these proteins lack clear homology to known proteins and are therefore regarded as taxon specific traits. Their detection opens the door for follow-up studies regarding their venomous function and origin. Our findings are setting the foundation for the study of nematocyst venom and its evolution in a rising lab model species with established experimental tools rarely available for a venomous animal.

## Electronic supplementary material

Below is the link to the electronic supplementary material.

Supplementary Figure 1 Expression patterns of *NvTld* (Tolloid) in *Nematostella*. In situ hybridization (ISH) was used in order to localize the expression, which was concentrated mostly in endodermal cells throughout the life cycle, as can be seen in the early planula (A: 2 days old), late planulae (C: 5 days old) and primary poly (B: 7 days old). In all panels the oral end lies to the right. (DOCX 1309 kb)

Table S1 (XLSX 21 kb)

Table S 2 (DOCX 12 kb)
