# Supplementary material for: Analysis of Soluble Protein Contents from the Nematocysts of a Model Sea Anemone Sheds Light on Venom Evolution
Source: Mar Biotechnol (NY). 2012 Nov 15;15(3):329–39. doi: 10.1007/s10126-012-9491-y (PMC3627010; doi:10.1007/s10126-012-9491-y)
Supplement: Supplementary file 1 — Expression patterns of NvTld (Tolloid) in Nematostella. In situ hybridization (ISH) was used in order to localize the expression, which was concentrated mostly in endodermal cells throughout the life cycle, as can be seen in the early planula (A: 2 days old), late planulae (C: 5 days old) and primary poly (B: 7 days old). In all panels the oral end lies to the right. (DOCX 1309 kb) [file 10126_2012_9491_MOESM1_ESM.docx]

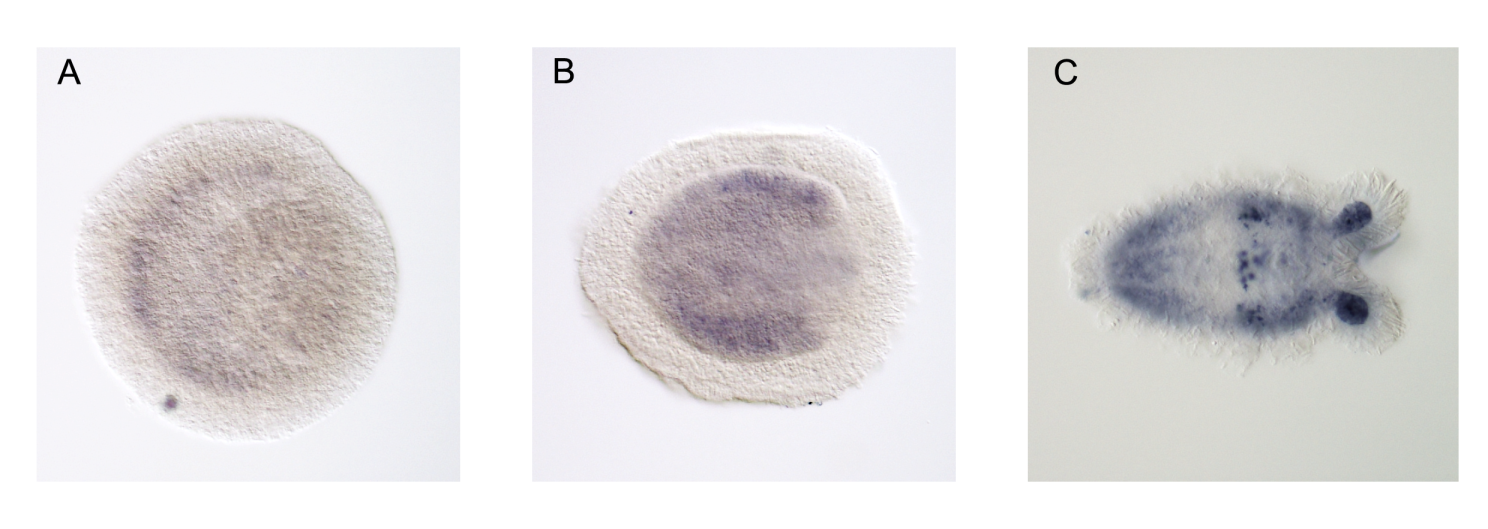


**Supplementary Figure 1.** Expression patterns of *NvTld* (Tolloid) in *Nematostella*. *In situ* hybridization (ISH) was used in order to localize the expression, which was concentrated mostly in endodermal cells throughout the life cycle, as can be seen in the early planula (A: 2 days old), late planulae (C: 5 days old) and primary poly (B: 7 days old). In all panels the oral end lies to the right.
