# Supplementary material for: Analysis of Soluble Protein Contents from the Nematocysts of a Model Sea Anemone Sheds Light on Venom Evolution
Source: Mar Biotechnol (NY). 2012 Nov 15;15(3):329–39. doi: 10.1007/s10126-012-9491-y (PMC3627010; doi:10.1007/s10126-012-9491-y)
Supplement: Supplementary file 3 — (DOCX 12 kb) [file 10126_2012_9491_MOESM2_ESM.docx]

| Primer | Usage |
| --- | --- |
| 5’- GATAACTTAGAGCTGGTTGAAGATG-3’ | Amplification of a fragment of NEP-6 |
| 5’-CGCCGTCCTTATCTTCATCCTCGTCA-3’ | Amplification of a fragment of NEP-6 |
| 5’- CCATTCTGGTGCGCGAGATAAGCCTCT-3’ | 5’ RACE of NEP-6 |
| 5’- CTCCTTGGTCATCAGCATGTCATCTTC-3’ | 5’ RACE of NEP-6, nested primer |
| 5’- AGCGCCGGCGACATTCGTCAGACGAAC-3’ | 3’ RACE of NEP-6 |
| 5’-CAAGTGCAATGCTCAAGGCGATAGTG-3’ | 3’ RACE of NEP-6, nested primer |
| 5’- AAAGATAAGGATCACACCGCCAGGTTC-3’ | Amplification of a fragment of NEP-16 |
| 5’- AGATTGCTTATCGAAGCTGCCTTGCTC-3’ | Amplification of a fragment of NEP-16 |
| 5’-GCGATTTGGTCTTCAGCACCTTCCTTAC-3’ | 5’ RACE of NEP-16 |
| 5’-GGTGCCATGGTAGGGGTACTGCCTTCT-3’ | 5’ RACE of NEP-16, nested primer |
| 5’- AATGCACATACTACGTCGGGAGATAC-3’ | 3’ RACE of NEP-16 |
| 5’-AGCTGGAAACATGATCGGAGATTTCG-3’ | 3’ RACE of NEP-16, nested primer |
| 5’-GATGGCGTCGAAACTCATCCTCGGA-3’ | Amplification of a fragment of NvNcol-3 |
| 5’-GTTTAGACGGCGTTGGTAGTAGACCGT-3’ | Amplification of a fragment of NvNcol-3 |
| 5’-CATCTGTGGCCACAGAGTAACAGA-3’ | Amplification of a fragment of NvNcol-3 |

Supplementary Table 2: Primers used in the study
